# Pre-Kidney Transplant Screening for Coronary Artery Disease: Current Practice in the United Kingdom

**DOI:** 10.3389/ti.2021.10039

**Published:** 2022-01-07

**Authors:** Ailish Nimmo, Matthew Graham-Brown, Sian Griffin, Adnan Sharif, Rommel Ravanan, Dominic Taylor

**Affiliations:** ^1^ North Bristol NHS Trust, Bristol, United Kingdom; ^2^ University of Leicester, Leicester, United Kingdom; ^3^ University Hospitals of Leicester NHS Trust, Leicester, United Kingdom; ^4^ Cardiff and Vale University Health Board, Cardiff, United Kingdom; ^5^ Queen Elizabeth Hospital Birmingham, Birmingham, United Kingdom

**Keywords:** kidney transplantation, screening, cardiovascular disease, survey, risk factors

Dear Editors,

Randomised control trial (RCT) evidence is not available to guide screening for asymptomatic coronary artery disease before kidney transplantation [1]. United Kingdom observational data show no clear benefit from screening [2]. To gain data representative of current practice in the United Kingdom, we invited a lead transplant nephrologist from each kidney transplant centre to complete a survey examining cardiac screening practice, work-up pathways, and appetite for a national RCT in June 2021. Ethical approval was not required.

Responses were received from all 23 (100%) centres, of which 22 had a protocol for cardiac assessment prior to listing. In three centres, asymptomatic individuals were not required to undergo cardiac investigation beyond an ECG or echocardiogram prior to transplantation. The remainder followed a risk-stratified approach; no centres performed universal screening.

In centres adopting risk-stratified screening, factors used to screen patients included a history of ischaemic heart disease (100% of centres), diabetes (100%), peripheral vascular disease (50%), smoking (50%), stroke (35%), limited exercise capacity (35%), hyper/hypotension (15%), or an abnormal echocardiogram (95%) or ECG (70%). Two centres stratified using the Newcastle Risk Index [3]. Thirteen centres had a specific age threshold (mostly 50 or 60 years), whilst others included age in combination with additional risk factors or Newcastle Risk Index scores.

The most frequent screening investigation was a myocardial perfusion scan (55%) followed by stress echocardiogram (20%). Coronary angiography and cardiopulmonary exercise testing were the initial investigation in one centre each. Other indications for coronary angiography included an abnormal initial screening test (39%) or on cardiology advice (35%). In one third of centres, the waiting time for investigations was over 10 weeks.

Nine centres had cardio-renal multidisciplinary meetings, whilst 14 had a designated cardiologist providing transplant candidate assessments. In 16 centres cardiology review was only needed for patients with abnormal screening tests, whilst in three cardiologists reviewed all screened patients.

Of 23 centres, 10 had updated their screening protocol within the past 2 years and three were in the process of an update. Whilst 19 centres reported experience of patient declines from listing based on an abnormal screening test, this amounted to one patient per month or less in 11 centres.

Respondents commented on the challenges of outdated evidence, reliance on observational data, and differences between real-world cohorts and RCT study populations when assessing the evidence for cardiac screening. The importance of cardio-renal meetings was noted in units not adopting screening. Of 23 centres, 22 expressed interest to participate in an RCT to examine the utility of screening, 12 of whom supported recruiting the highest cardiac risk candidates.

Our survey highlights variation in screening practice across the United Kingdom (Figure 1). Similar heterogenous practice has been shown in the United States [4], although our survey was undertaken following publication of ISCHEMIA-CKD [5]. Whilst no centres perform universal screening and many have recently updated their protocols, which may represent a trend away from routine screening, responses highlight nephrologists’ concerns over the evidence upon which practice is based. Capturing views of other transplant professionals and patients is essential, but this survey suggests support for an RCT to evidence utility of screening.

**FIGURE 1:**
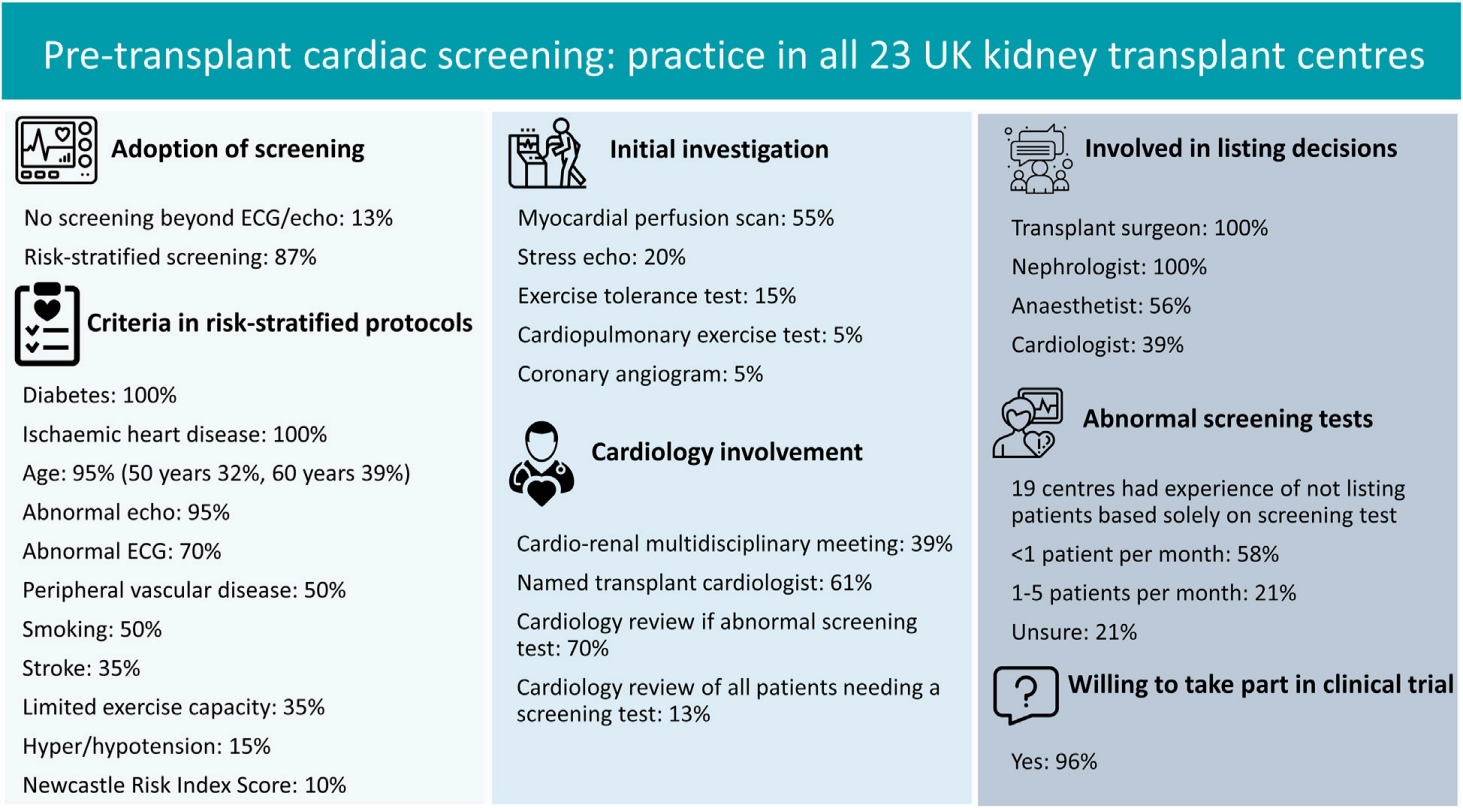
Summary of findings from survey of pre-transplant cardiac screening.

## Data Availability

The raw data supporting the conclusions of this article will be made available by the authors, without undue reservation.

## Abbreviations

ECG, Electrocardiogram; RCT, Randomised controlled trial.
